# Perceptions of Pregnant Women on Traditional Health Practices in a Rural Setting in South Africa

**DOI:** 10.3390/ijerph19074189

**Published:** 2022-04-01

**Authors:** Mvulakazi Patricia Thipanyane, Sibusiso Cyprian Nomatshila, Olanrewaju Oladimeji, Hannibal Musarurwa

**Affiliations:** 1 Preventive Medicine and Health Behavior Unit, Department of Public Health, Faculty of Health Sciences, Walter Sisulu University, Mthatha 5117, South Africa; snomatshila@wsu.ac.za; 2 Community Medicine Unit, Department of Public Health, Faculty of Health Sciences, Walter Sisulu University, Mthatha 5117, South Africa; ooladimeji@wsu.ac.za; 3 Department of Human Biology, Faculty of Health Sciences, Walter Sisulu University, Mthatha 5117, South Africa

**Keywords:** traditional practices, culture, pregnant women, perceptions, qualitative study

## Abstract

Though there are prenatal and perinatal protocols across the African continent, traditional practices are commonly used. Improving maternal health requires an understanding of local cultural approaches and traditional health systems. The purpose of this study was to determine the perceptions of pregnant mothers of various ages and gravidities towards traditional maternal health practices during pregnancy. A descriptive phenomenological study was carried out with 27 purposively selected pregnant women from the King Sabatha Dalindyebo health sub-district. Discussion from three focus groups yielded three major themes and eight sub-themes. Content analysis revealed strong opinions and support for traditional practices. Although specialist practitioners were mentioned, family members were the primary source of information on traditional perinatal health practices. African mahogany bark, herbal decoctions, and holy water were allegedly used to treat labor pains, postnatal care, and the warding off of evil spirits. During pregnancy, these were thought to be beneficial and necessary. Nonetheless, the protection of some traditional practices’ know-how prevents integration of the traditional health system into the national health system. Consequently, there is a need for dialogue to facilitate the exchange of ideas on maternal health between the two health systems in order to facilitate more efficient policy formulation and implementation.

## 1. Introduction

Traditional medicine is used by more than 80% of the people in Africa, with the majority believing it is the only accessible primary health care option, especially in rural areas where a variety of traditional medicines, including herbal medicines, are used [1,2,3]. It is reported that the usage of herbal medicine during pregnancy varies a lot depending on where you live, your ethnicity, your cultural customs, and your socioeconomic level [4]. The World Health Organization (WHO) defines traditional and complementary medicine (TCM) as a broad set of health-care practices, approaches, knowledge, and beliefs that include plant-, animal-, and mineral-based medicines; spiritual therapies; manual techniques; and exercises, all of which can be used alone or in combination to treat, diagnose, and prevent illness or maintain well-being [5]. Traditional medicines are further reported to be contributing immensely to therapeutic purposes such as management of inflammation, coughing, antipyretic and various other ailments regardless of place of residence, educational level, and sources of income [6,7,8]. This is despite the fact that the safety and security of the preparation processes for these practices is unknown to the general public [9]. However, cultural and traditional health practices are reported to be playing an important role in maternal health care in most parts of Africa, with traditional remedies used by pregnant women for a variety of reasons [10,11]. It is also a common practice in Africa that people often hold to the traditional assumption that traditional birth attendants are the only ones who can help with pregnancy and delivery in their spaces [12]. Both developing and developed countries experience use of traditional medicines by pregnant women. For instance, in a study conducted in Saudi Arabia in 2017, use of traditional medicines was reported by 335 pregnant women [13]. Similarly, in Malaysia, use of herbal medicines was reported by 34% of pregnant women [14]. Their use was reported by 32% of pregnant women in Central Africa, while 45% reported their use in East Africa [15]. Though studies have shown that use of herbal medicine was prevalent among pregnant women across sub-Saharan Africa, there is a scarcity of information on its use in the region [15,16].

Herbal or traditional medicine is thought to be a factor in inadequate access to and usage of maternal healthcare services, such as prenatal care and health-care facility delivery, which are targeted at reducing maternal mortality [17]. As a result, traditional medicines are becoming a topic of rising public health concern in the developing world, particularly in Africa [18]. This is exacerbated by pregnant women’s failure to report traditional medicines when questioned by the health officials who treated them at the health facilities [4,19]. As a result, the lack of transparency, combined with the unknown composition of traditional medicines, may cause harmful side effects in patients, as well as have an impact on maternal and child health during pregnancy, which are import indicators for women’s health [16,20].

Pregnant women use traditional health medicines to have a hassle-free pregnancy, to shorten labor, and to improve breastfeeding [13]. Thousands of women encounter obstetric difficulties every year, the majority of which are caused by haemorrhage, puerperal infection, obstructed labor, gestational hypertension, maternal sepsis, and illegal abortions [21,22]. Medication, herbs, and supplements are usually linked to such adverse effects among pregnant women; thus, they should be strictly avoided [23]. Most health care providers are unaware of the use of herbal medicines in pregnancy, which poses a significant difficulty for them during time when they have to offer care and puts pressure on the national health system [24,25]. Herbal medicine toxicity issues among users can be linked to a lack of sufficient standardization and quality control in the official herbal sector, in addition to the use of intrinsically hazardous medicinal herbs [26,27].

Understanding the relationship between local cultural traditions and women’s perceptions of perinatal care may aid in the development of better prenatal and delivery services, especially in rural areas where access to quality antenatal and birth care is limited [19]. The purpose of this study was to determine the perceptions of pregnant mothers of various ages and gravidity towards traditional maternal health practices during pregnancy.

## 2. Materials and Methods

### 2.1. Study Design, Setting, Population, and Sampling 

The researchers used a descriptive phenomenological qualitative research method for the study.

This study was conducted in King Sabata Dalindyebo (KSD) sub-district, which has forty-nine clinics and five Community Health Centers. These health facilities provide antenatal care to a community of pregnant women who make up 488 349 people who live in rural and urban locations of the sub-district [28].

Pregnant women of any gestational period aged 18 year and above, residing in KSD subdistrict, were members of the population and were therefore committed to carrying a healthy baby throughout their pregnancy. This commitment would lead pregnant women to seek out any assistance or practice that they believed would benefit their health and that of their babies. Pregnant mothers participated in three focus group discussions (FGDs) (FGD-A, FGD-B, and FGD-C). Non-pregnant women and pregnant women not residing in study setting were excluded as they were considered as lacking experience on studied topic. Purposive sampling was used to select participants for focus groups. The FGD-A had twelve people, the FGD-B had nine, and the FGD-C had six. These Focus Group Discussions were held within premises of the health facilities in KSD. The KSD sub-district was chosen because the South African National Department of Health identified it as one of the areas with poor maternal outcomes in 2013.

### 2.2. Data Collection 

All participants were given the chance to participate in focus group discussions (FGDs) to learn more about how pregnant women in the KSD sub-district perceive traditional health practices. Participants were selected with intention to cover homogeneity without considering any level of social clustering. The FGDs were conducted at the health facilities at times convenient to participants between October and December 2017. As a result of data saturation, a total of three focus groups were held, each lasting no more than 90 min. The conversations were facilitated by a public health and health promotion specialist who remained neutral and observed passiveness and dominance among participants during discussions. Semi-structured schedules with open-ended probing and follow-up questions were prepared for collection, as shown in Table 1. All focus group conversations took place in IsiXhosa, a widely spoken local language. Discussions were only audio-recorded in accordance with participants’ preference and were professionally documented by a competent transcriptionist for interpretation. The participants were asked for their permission to record the FGD conversations. Sociodemographic characteristics for the participants were also collected during FGDs.

### 2.3. Data Analyses

Descriptive data from the FGDs were analyzed immediately after FGDs using the content analysis approach [29]. To avoid misinterpretation of key information, all data collected from the sound recorder were meticulously transcribed. The audio recorder was started and stopped several times to accurately capture substance in the recordings, whilst data from field notes were recorded and included within transcription. To avoid distortion, forward and backward translation from isiXhosa to English and back to isiXhosa were carried out. Peer checking of all three transcripts was done by second and third authors. Themes and sub-themes were created from categories with comparable meanings, and the themes were confirmed using an independent coder. The coding and grouping were done using a test-retest method. Following the completion of all transcribing operations, sound recordings were deleted in accordance with confidentiality guidelines.

### 2.4. Ethics and Consent 

The Walter Sisulu University’s Human Research Ethics and Biosafety Committee granted ethical approval (016/16) for the study. Permission to use health facilities was granted by the Eastern Cape province’s Departments of Health with permission number EC_2017RP38_330. Written informed consent with full details about the study and reporting channels was sought from the participants. All research transcripts were kept in a locked filing system with all electronic data coded and secured with a password-protected filing system. Participants’ identities were not recorded or disclosed in any way.

### 2.5. Trustworthiness

The study’s credibility, confirmability, transferability, and dependability were all tested in accordance with Guba et al. findings [30]. In order to grasp the participants’ perspectives on the utilization of traditional practices, the researcher had to spend more time with participants. The researcher was able to collect more data through probing and questioning led by the flow of discussions. Tape recording and note capturing were effective elements for collecting and aligning a variety of perspectives and information in order to assure accuracy in recording responses.

## 3. Results

Table 2 indicates that participants from the three FGD were mostly (66.7%) aged 25–33 years, with majority (85.3%) having had 2–4 pregnancies in their lifetime. A total of 66.7% participants had secondary school education, whilst just above half (51.9%) were unemployed.

From a total of three FGDs, the data gathered for this study resulted in a total of 27 participants who were pregnant women in KSD sub-district of Eastern Cape province, South Africa. There were 12 participants in FGD-A, nine in FGD-B, and six in FGD-C. Table 3 indicates three themes and eight sub-themes that emerged from the data.

### 3.1. Theme 1: Sources of Information 

#### 3.1.1. Subtheme 1.1: Family Members

Participants reported that their family members throughout all ages played an important role in sharing information about the use of traditional health practices during practices. One participant said,

*“My mother gave me a lesson during my first pregnancy about traditional medicines used when pregnant”.*

Participant 4 from FGD-A

*“After getting married, my eldest sister- in- law told me about the importance of using traditional medicines so as to conceive faster”*

Participant 3, FGD-B

*“I am staying with my grandmother who recommends herbal decoction claiming that it helped her a lot when she was pregnant with my mother”*

Participant 1, FGD-C

#### 3.1.2. Subtheme1. 2: Community 

The participants reported that they were made aware by the community about the traditional practices and products used by women during pregnancy. Participants said:

*“We hear about traditional health services from the community people who could tell you where to go for any pregnancy related problem”*

Participant 5 from FGD-A

*“I am pregnant for the first time, but I have been told by other people in the community about the good work done by herbal decoction to a pregnant person”*

Participant 1, FGD-B

*“I never got married but I was pregnant when I was at still at my parent’s home. Through communicating with other elderly women in the community, I am now aware of traditional practices and medicines used during pregnancy”*

Participant 5 from FGD-A

#### 3.1.3. Subtheme1. 3: Other Pregnant Women

Peers and other pregnant women were also reported to be playing significant role in recommending and supporting use of traditional health services.

*“When I attended the antenatal clinic, I heard other pregnant women talking about African mahogany tree bulk product which is applied to the baby’s face. It clear baby rash and the fine hair on the baby’s skin “*

Participant 3, FGD-C

*“All pregnant women I have met recommended traditional concoction for curing traditional illnesses like diarrhea during pregnancy and after delivery”*

Participant 2 from FGD-C

### 3.2. Theme 2: Providers of Traditional Health Services 

#### 3.2.1. Subtheme 2.1: Faith-Based 

Among the number of traditional health service providers, faith-based healers were identified as some of providers whose services included prayers, cords, and holy water. Some participants reported that they did not believe in use of traditional medicines but believed in prayers. Such participants used faith-based healers for the service as they aligned better with their belief system.

*“There is a cord which is prayed for that you get from St John’s church, and you tie it around the waist when you are pregnant”*

Participant 10 from FGD-A

*“There is another cord which is made from wool, and you get it from faith a healer or the herbalists. It depends on what you believe in”*

Participant 1 from FGD-A

*“I don’t believe in traditional medicines like herbs and mixtures, I am using holy water and it works wonders for me in all my pregnancies”*

Participant 1 from FGD-B

#### 3.2.2. Subtheme 2.2: Herbalists 

Herbalists were reported as another group of providers who were considered to have ability to prevent any traditional illnesses for babies, and for mothers even when they were still pregnant. Herbalists were perceived as being skilled at preventing illnesses among those who complained about them, and were further skilled at cleaning the womb.

*“When you go to a herbalist, you are given a concoction prepared from different medicines from the chemist”*

Participant 9 from FGD-A

*“Some traditional practitioners provide a traditional concoction to the pregnant woman to cure traditional illness like diarrhea in the unborn baby and this is also given to the baby after delivery”*

Participant 2 from FGD B

*“There are traditional activities done by herbalist to prepare a pregnant woman, especially if you had miscarriages each time you fell pregnant”*

Participant 3 from FGD-A

*“Herbalists provide herbal medicines in the form of powder or mixture to pregnant women”*

Participant 9 from FGD-A

*“The elders took me to a herbalist for herbal decoction to have my womb cleansed in preparation of conception”*

Participant 5 from FGD-B

#### 3.2.3. Subtheme 2.1: Other 

Other participants indicated that they prepared their own traditional medicines from the knowledge accumulated from experience using ingredients from traditional medicines pharmacies or from fauna and flora. These concoctions were perceived to be playing important roles in preventing complications during pregnancy and encouraging milk flow after giving birth.

*“You get herbal decoction from the Pharmacy as a ready to use mixture”*

Participant 8 from FGD-A

*“Herbal decoction is either bought as traditional concoction or as herb which you prepare yourself when you are pregnant”*

Participant 12 from FGD-A

*“I used herbal decoction with my previous pregnancies, and I never had any complications, and even with this pregnancy I am using it”*

Participant 7 from FGD-B

*“There is a black powder called creosote which is applied on the nipples after delivery to encourage the flow of breastmilk”*

Participant 9, FGD-B

*“Herbal decoction is prepared by an elderly woman for the pregnant woman who will drink it daily”*

Participant 3, FGD-C

*“My sister had a problem of having pregnancy miscarriages until she used herbal decoction. Fearing that I might have the same problem, I started using it also with the current pregnancy”*

Participant 5, FGD-C

### 3.3. Theme 3: Effects of Traditional Health Practices Used during Pregnancy

#### 3.3.1. Subtheme 3.1: Positive Effects of Traditional Practices Used during Pregnancy

Various effects perceived to be positive were reported by the participants about the use of traditional health practices. These included protection from evil spirits, improved fertility, reduction of labor period, prevention of acid reflux, softening of the abdomen to enable easy kicking by the baby, and prevention of complicated invasive deliveries.

*“There are evil spirits which attack us when we are pregnant, and we therefore use traditional medicines to protect ourselves and our unborn babies from them”*

Participant 12, FGD-A

*“Herbal decoction is mostly used by pregnant women so that the baby does not have traditional illness (diarrhea) after delivery. This comes as traditional concoction which you drink until you deliver the baby “*

Participant 7, FGD-A

*“When you are pregnant you worry a lot about getting miscarriage, and in order to prevent it you use herbal decoction or herbal decoction”*

Participant 8, FGD-A

*“If you use herbal decoction during pregnancy, you deliver the baby easily and fast without complications or operation…”*

Participant 10, FGD-A

*“A pregnant woman does not eat everything like, acidic and fatty foods because she will have heartburn and when she gives birth to the baby, she will have eyes with discharge”*

Participant 1, FGD-A

*“Herbal decoction helps to soften the abdomen of a pregnant woman if it happens to be tense so that the baby kicks well”*

Participant 3, FGD-A

*“If you used herbal decoction when you were pregnant, you would not have prolonged labor leading to theatre”*

Participant 8, FGD-A

*“Herbal decoction helps a pregnant woman not to have obstructed labor which often leads to caesarean section”*

Participant 6, FGD-B

*“I have seen herbal decoction being good in causing the infertile woman to conceive; and protecting the baby from having traditional illness like diarrhea after delivery “*

Participant 3, FGD-B

*“Herbal decoction cleanses the body of the woman if she is taking a lot of acidic foods during pregnancy”*

Participant 4, FGD-B

*“Castor oil mixed with ‘hermensis’ is used on the abdomen of a pregnant woman to protect the unborn baby from the evil spirits, and also helps the woman from having constipation”*

Participant 5, FGD-B

*“It is wise to take herbal decoction towards the end term of pregnancy because it softens the abdomen and facilitates labor”*

Participant 2, FGD-B

*“Herbal decoction helps the woman not to have obstructed labor which often leads to caesarean section”*

Participant 2, FGD-C

*“If a woman is infertile and is given herbal decoction, she becomes pregnant after using it”*

Participant 5, FGD-C

*“We hear that if a pregnant woman uses herbal decoction, she does not experience problems when she is about to deliver the baby”*

Participant 4, FGD-C

#### 3.3.2. Subtheme 3.2: Negative Effects of Traditional Practices Used during Pregnancy

Participant identified some negative effects from the use of traditional products during pregnancy, indicating that these are sometimes too strong for unborn babies to tolerate, and babies are often born looking older than their age. Participants further reported that some traditional practices have tendency of causing stillborn deliveries,

*“Although we say herbal decoction is good to the baby, but it is very dangerous because it causes very strong and irregular contractions which may kill the baby or rupture the uterus of the mother”*

Participant 11, FGD-A

*“A mother who used herbal decoction usually gives birth to a baby who looks old because of the wrinkles which are caused by herbal decoction”*

Participant 7, FGD-A

*“When herbal decoction is used as an enema it depresses the frontal fontanelle of the baby, and this often results to the baby dying”*

Participant 3, FGD-B

*“I am not sure how herbal decoction is prepared now because most pregnant women who use this product have ruptured uterus, others are taken to operating theatre for delivery of their babies”*

Participant 1, FGD-B

*“The disadvantages are usually noticed in the clinic or hospital when the woman in labor is having complications …”*

Participant 2, FGD-C

## 4. Discussion

The study sheds light on the use of traditional medicinal practices by pregnant women in the King Sabata Dalindyebo sub-district. There are different traditional products that are used by pregnant women that originate from cultural and religious beliefs. Concerns and fears about the safety and health of the pregnant mother and the baby, protection from metaphysical forces, and socio-cultural beliefs were the main drivers for using traditional medicines.

The fear of being attacked by evil spirits during pregnancy is greater than the fear of being affected by natural causes such as diabetes, asthma, or hypertension, because the supernatural evil spirits are believed to cause serious pregnancy-related complications such as miscarriage, abnormal presentation of the baby in the uterus, malformed baby, stillbirth, and the death of the mother while pregnant. The culture, beliefs, and perceptions of the different clans and communities about pregnancy shape traditional practices used during pregnancy. Likewise, in other studies, it was indicated that the culture and beliefs of the communities influenced and underpinned the traditional health-seeking behavior of a woman during pregnancy [31,32]. It was also discovered that when indigenous practices were used by the people of the former Transkei, pregnant women in this rural area depended on the natural plant resources of their environment for medicine; food; and pastoral, cultural, and religious care, during pregnancy [33]. Other authors also confirm that indigenous medicinal plants contribute significantly to a large portion of the South African population that lacking easy access to healthcare services, and that these medicinal plants are found in the informal market places [34].

This study has shown that traditional medicinal products were used during pregnancy regardless of pregnant women’s general awareness, knowledge, and perceptions, as well as their ages, gravidity, level of education, and employment. These findings were consistent with previous research, which found that pregnant women with low education, low income, and who lived far from health facilities were much more aware of traditional medicines used during pregnancy than those with a high level of education and income [25]. As a result, knowledge of herbal medicine use among pregnant women in rural areas, as well as the potential negative effects of numerous herbs during pregnancy, was found to be limited in other setting of similar nature [35]. The association between the use of indigenous practices by pregnant women and socioeconomic and demographic characteristics of the participants had community-based cultural meanings [26]. The employment levels of women did not influence the knowledge of pregnant women about the traditional medicinal products they use during pregnancy. Contrary to the findings of this study, other researchers found that the users of traditional medicinal plant products are more likely to be of low socio-economic and educational status because they can afford to pay for them. This is in contrast to western medicinal products, which are expensive [19]. These researchers further state that the formal natural products benefit those people with better socio-economic standing, more so than the informal products, which benefit people of low socio-economic status.

This study revealed that more than 11% of women who were pregnant for the first time knew about the same traditional products, and this was confirmed by another study that had similar findings: the use of traditional medicinal products by pregnant Zimbabwe women was not significantly associated with nulliparity and null gravidity [36]. This resulted in pregnant women having to rely on advice from elderly women in the community as well as from traditional health practitioners about the health products used during their pregnancy.

Traditional health products were used to protect the mother and the baby from any perceived causes of ill-health that could affect them physically, psychologically, spiritually, or socially. Such products included holy water, cord prayed for, herbal decoction, traditional illness (diarrhea), and holy water. According to Peltzer et al., an egg of a slaughtered hen would be broken on the head of a pregnant woman so as to release the woman from any magic and evil spirits that were attacking her and the baby [37]. Although all pregnant women have the same desires and concerns for a healthy and safe pregnancy, the conventional methods they follow are not the same. Unlike the women in this study, Yoruba women in Nigeria put safety pins on their abdomens when they were pregnant to keep evil spirits out of the uterus that was carrying a baby. They stayed out of the sun because they were afraid of being harmed by evil spirits or ghosts. They did not go out at night because they were afraid the child might be replaced by an evil spirit or be misshapen. The researchers also discovered that pregnant women avoided eating rodents, snakes, and snails for fear of the baby crawling on their chests, as well as avoiding stealing when pregnant to avoid the infant being kidnapped [31].

Similar observations were made in Ghana, where there were prohibitions such as not strolling near graveyards, not going out at specified times of the day, and not mingling with people who were considered to be wicked [38]. Also reported regarding maternal health practices, beliefs, and customs in Southeast Madagascar was the fact that pregnant women did not consume anything bitter, to prevent the baby from having stomach cramps and the pregnant woman from experiencing heartburn [12].

Religious beliefs and practices, as indicated in this study, were reported to be playing an important role among pregnant women in Ghana, who did not believe in traditional herbs; instead, they consulted faith-based healers who gave them ‘some stuff’ and prayed for it to be used during pregnancy [38]. Pregnant women are said to express their religion by praying, singing, dancing, expressing gratitude in church, and expressing fellowship. The spiritual healer would give the pregnant women revelations, reverse negative dreams, lay hands on them, and anoint them. During pregnancy, there were restrictions on food, water, and tribal ceremonies. They used the Holy Bible, anointed oil, blessed water, sticker, blessed white handkerchief, blessed sand, and Rosary during their pregnancy.

Residing within the same community that observed its culture and tradition did not make everyone within that community comply with the culture and tradition. There were also mothers, as much as they were residing within the same community of the study, who were not clear about the traditional practices used during pregnancy. This lack of knowledge was due to the different belief systems that people had; those who were inclined in western medicinal practice would disregard traditional practice and vice versa. Participants reported restrictions on fatty food, fizzy drinks because they cause heartburn, and giving birth to a baby with discharging eyes. The same observation was made in Zambia, where pregnant women believed having a balanced diet such as eggs, okra, bones, offal, sugar cane, and salt was associated with giving birth to healthy babies [39].

Pregnant women sought advice from a variety of traditional health practitioners, who offered a variety of services and used a variety of traditional health products. They sought the help of divine and faith-based healers, who prayed for them and gave them holy water. They made traditional products for the mothers as well as providing prenatal care. Traditional medicinal products were either provided through divination, where information beyond the reach of the rational mind is accessed by using cowry shells; through the throwing of bones, shells, money, seeds, dice, and flat pieces of wood to diagnose and treat illness; or through spiritualism using prayers, where faith-based providers consult God through prayer to guide and give direction about the conditions presented to them [27]. The traditional methods and products were reported by participants in the current study as being used for cleaning the uterus so as to prepare it for conception; facilitating conception in women who have infertility problems; and correcting abnormal presentations, such as breech and transverse lie of the baby in the uterus. These products were also used for curing traditional illnesses (diarrhea) in the baby whilst in utero so that she would not have traditional illnesses (diarrhea) after birth, for inducing labor, and for ensuring a complication-free delivery. Similar findings were documented in the Middle East, where traditional medicines were commonly used during pregnancy for treatment of gastro-intestinal disorders, common colds, and flu because they had fewer side effects than western medicines, highlighting cultural differences between South African and Middle Eastern mothers [3].

In North-East Scotland, complementary and alternative medicine is also practiced during early pregnancy. There were over twenty-eight alternative medicine modalities administered, with oral medicines being the most commonly used [40]. Nausea, urinary tract symptoms, digestion, labor preparation, constipation, skin disorders, and dental difficulties were among the cited reasons for practicing these techniques. The use of traditional products was also reported in the current study to produce positive results. For instance, participants reported that infertile women would conceive; a baby who presented as a breech would be corrected to normal cephalic presentation; a woman who had passed term of pregnancy and was therefore overdue was given a herbal decoction to induce labor; and a woman who was using traditional products would not choose a caesarean section. Traditional medicines or remedies were commended by participants in another study where it was reported that they were commonly used by pregnant Batswana women of South Africa for orally to induced labor [41].

Despite the fact that pregnant women utilized these medications, they were unsure about their safety, effectiveness, or potential drug interactions when used with prescription medications obtained from a health facility. These safety issues were confirmed in the current study, where participants indicated that they were not sure about the preparation procedure and were aware of complications such as raptured membranes, complicated deliveries, and delivery of old-looking babies among those who used traditional medicines. These findings are in line with findings in the study conducted in Saudi Arabia [13]. This was exacerbated by the fact that most traditional medicines were mixed and stored in bottles that seldom had any expiry date or dosages written on them. It can therefore be easy for a pregnant woman to take the medicine as much as she wants, depending on the desired results of the medicine taken. This practice was perceived to have the potential to lead to drug-related complications like poisoning, intoxication, and reactions. Although there was a popular belief that when a pregnant mother took herbal decoction, she would not have prolonged labor and complicated delivery, most of these women who took this product were reported to have presented in the hospital with obstructed labor, ruptured uterus, and babies having asphyxia. Similar complications following the use of herbal medicines were also reported in other studies, which recorded that these medicines were toxic to the embryo and caused defects of the baby, teratogenic effects, and premature labor [4]. This was also supported by additional research that found that the most common side effects of phytomedicines were a lack of newborn respiratory drive, neonatal mortality with abnormal fetal heart rhythms, and placenta abruption [42]. Both the mother and the infant experienced oxytocic complications, as well as the bright green meconium passed by the newborn.

Safety concerns of these products and practices of the mother and the baby were also shared, which were drug interactions if the mother used traditional products concurrently with western medicine, as well as the possibility of potential drug intoxication of both the mother and the baby [4]. As such, according to the literature, randomized clinical trials and tests to confirm the safety of these traditional herbal medicines are urgently needed to validate their quality [35]. This is supported by a study that uncovered harmful and forceful traditional practices such as putting Lao women on a “hot bed” after delivery, where a fire was lit beneath the bed of a woman who had just given birth. Salt would be added to the fire, which was thought to help the newborn’s eyesight and the woman’s health after delivery, as well as encourage uterine contractions [43]. These practices have the potential to cause health risks such as burns, hyperthermia, brain damage, epileptic seizures, and unsuccessful breastfeeding, which would in-turn put a strain on the overly stretched health system. Similar observation was made in a similar study where the researcher looked at ‘roasting and steaming’ of pregnant women in Cambodia, which involved the mother lying above a bed of burning coals after giving birth in order to keep her warm and restore energy [44]. This roasting of the mothers was done from three days to one month after delivery and this could be harmful and fatal to the mother.

Same as in King Sabata Dalindyebo Sub-district, the strong traditional beliefs and practices found in Maharashtra led to the poor utilization of maternal and child health services during pregnancy and delivery, resulting in late prenatal bookings. Such poor maternal and child care services often lead to unhygienic cord care practices, which can cause tetanus and septicaemia to the baby; delay in initiation of breastfeeding involved depriving the new-born of very nutritious and essential colostrum [45].

This study was able to identify the extent to which pregnant women in the KSD subdistrict use traditional medicines and how this affects their use of the mainstream health system, hence affecting maternal health outcomes in general. This research has also shed light on traditional behaviors followed by pregnant women, indicating the necessity for more research in the future.

## 5. Limitations 

The authors made a significant effort to delimit the study. However, this study still experienced some limitations. The study focused only on pregnant women in selected facilities in the sub-district. As a result, it is not generalizable. The nature of the study, as a qualitative study, means that reporting was based on self-reported perception. Fewer participants were enrolled, subjecting the study to underreporting. The study was also limited by the scarcity of recent literature on the studied topic in South Africa.

## 6. Conclusions

Pregnant women in King Sabata Dalindyebo sub-district use traditional health products and practices due to cultural and religious beliefs. Participants perceived the use of these products and practices as being important for protecting the mother and baby from evil spirits and ensuring a smooth pregnancy and delivery. This demonstrated the level of risk that mothers face when the traditional health space is not tightly controlled and monitored, and demonstrated the level of risk that mothers face when the traditional health space is not tightly controlled, monitored, or even supported by science. This study concludes that the use of both traditional health products and conventional medicine models demonstrates the need for a functional integrated public health system that encompasses both systems used by the population. As a result, policy reform and the strengthening of the existing framework for integrating traditional health services into the conventional health system require special attention. A clear recognition of the traditional health system could lead to a better understanding of the nature of the products used, preparing health professionals to respond in times of need. This would give the space even more control and ensure that desirable maternal outcomes were achieved at all times.

## Figures and Tables

**Table 1:** Sample of major questions asked during FGDs.

Subject	Question
Information about traditional health practices	What do you understand about traditional health practices?
	What are your sources of information on these?
Traditional practices	What are your perceptions on the use of traditional health practices during pregnancy?
	What do you think is the role of traditional health practices during pregnancy?
	What do you think are the effects of these in maternal outcomes?

**Table 2:** Demographic characteristics of the participants.

Variables	Categories	Frequency (n = 27)	Percent (%)
Age groups	18–24 years	5	18.5
	25–33 years	18	66.7
	35 and above	4	14.8
Gravidity	2–4 pregnancies	23	85.2
	5–8 pregnancies	1	3.7
	Other	3	11.1
Educational level	Secondary	18	66.7
	Post matric	9	33.3
Employment status	Unemployed	14	51.9
	Employed	13	48.1

**Table 3:** Themes and sub-themes emerged for the data.

Theme	Subthemes
1. Sources of information	1.1 Family members
	1.2 Community members
	1.3 Other pregnant women
2. Providers of traditional health services	2.1 Faith-based
	2.2 Herbalists
	2.3 Other
3. Effects of traditional practices used during pregnancy.	3.1 Positive effects of traditional practices used during pregnancy
	3.2 Negative effects of using traditional products during pregnancy

## Data Availability

Data are available on request, though thay will be guided by research regulations, Protection of Personal Information Act, and the confidentiality agreement with participants.

## References

[1] Kasilo O.M., Trapsida J.-M. (2010). Regulation of traditional medicine in the WHO African region. Afr. Health Monit. (Online).

[2] Lu Y., Hernandez P., Abegunde D., Edejer T. (2011). The World Medicines Situation 2011.

[3] John L.J., Shantakumari N. (2015). Herbal medicines use during pregnancy: A review from the Middle East. Oman Med. J..

[4] Illamola S.M., Amaeze O.U., Krepkova L.V., Birnbaum A.K., Karanam A., Job K.M., Bortnikova V.V., Sherwin C.M., Enioutina E.Y. (2020). Use of herbal medicine by pregnant women: What physicians need to know. Front. Pharmacol..

[5] Qi Z., Kelley E. (2014). The WHO traditional medicine strategy 2014-2023: A perspective. Science.

[6] Golmei P., Kalita P., Agrawal S., Ahmed A.B., Sen S., Chakraborty R. (2021). Pharmacovigilance: Methods in Developing the Safety and Acceptability of Traditional Medicines. Evidence Based Validation of Traditional Medicines.

[7] Gyasi R.M., Asante F., Segbefia A.Y., Abass K., Mensah C.M., Siaw L.P., Eshun G., Adjei P.O.-W. (2015). Does spatial location matter? Traditional therapy utilisation among the general population in a Ghanaian rural and urban setting. Complement. Ther. Med..

[8] Mekuria A.B., Erku D.A., Gebresillassie B.M., Birru E.M., Tizazu B., Ahmedin A. (2017). Prevalence and associated factors of herbal medicine use among pregnant women on antenatal care follow-up at University of Gondar referral and teaching hospital, Ethiopia: A cross-sectional study. BMC Complement. Altern. Med..

[9] Fokunang C., Ndikum V., Tabi O., Jiofack R., Ngameni B., Guedje N., Tembe-Fokunang E., Tomkins P., Barkwan S., Kechia F. (2011). Traditional medicine: Past, present and future research and development prospects and integration in the National Health System of Cameroon. Afr. J. Tradit. Complement. Altern. Med..

[10] Mothupi M.C. (2014). Use of herbal medicine during pregnancy among women with access to public healthcare in Nairobi, Kenya: A cross-sectional survey. BMC Complement. Altern. Med..

[11] de Boer H., Lamxay V. (2009). Plants used during pregnancy, childbirth and postpartum healthcare in Lao PDR: A comparative study of the Brou, Saek and Kry ethnic groups. J. Ethnobiol. Ethnomed..

[12] Morris J.L., Short S., Robson L., Andriatsihosena M.S. (2014). Maternal health practices, beliefs and traditions in southeast Madagascar. Afr. J. Reprod. Health.

[13] Aljofan M., Alkhamaiseh S. (2020). Prevalence and Factors Influencing Use of Herbal Medicines During Pregnancy in Hail, Saudi Arabia: A cross-sectional study. Sultan Qaboos Univ. Med. J..

[14] Kim Sooi L., Lean Keng S. (2013). Herbal medicines: Malaysian women’s knowledge and practice. Evid. -Based Complement. Altern. Med..

[15] Ahmed S.M., Nordeng H., Sundby J., Aragaw Y.A., de Boer H.J. (2018). The use of medicinal plants by pregnant women in Africa: A systematic review. J. Ethnopharmacol..

[16] El Hajj M., Holst L. (2020). Herbal Medicine Use during Pregnancy: A Review of the Literature with a Special Focus on Sub-Saharan Africa. Front. Pharmacol..

[17] UDHS I. (2011). Uganda Demographic and Health Survey.

[18] Bodeker G., Kronenberg F. (2002). A public health agenda for traditional, complementary, and alternative medicine. Am. J. Public Health.

[19] James P.B., Wardle J., Steel A., Adams J. (2018). Traditional, complementary and alternative medicine use in Sub-Saharan Africa: A systematic review. BMJ Glob. Health.

[20] World Health Organization (2015). Maternal Mortality Fact Sheet No. 348.

[21] Olatunji A., Sule-Odu A. (2001). Maternal mortality at Sagamu, Nigeria—A ten year review (1988–1997). Niger. Postgrad. Med. J..

[22] Gülmezoglu A.M., Lawrie T.A., Hezelgrave N., Oladapo O.T., Souza J.P., Gielen M., Lawn J.E., Bahl R., Althabe F., Colaci D. (2016). Interventions to reduce maternal and newborn morbidity and mortality. Reprod. Matern. Newborn Child Health.

[23] Bercaw J., Maheshwari B., Sangi-Haghpeykar H. (2010). The use during pregnancy of prescription, over-the-counter, and alternative medications among Hispanic women. Birth.

[24] Adams J. (2011). Growing popularity of complementary and alternative medicine during pregnancy and implications for healthcare providers. Expert Rev. Obstet. Gynecol..

[25] Gaede B., Versteeg M. (2011). The state of the right to health in rural South Africa. S. Afr. Health Rev..

[26] Mensah M., Komlaga G., Forkuo A.D., Firempong C., Anning A.K., Dickson R.A. (2019). Toxicity and safety implications of herbal medicines used in Africa. Herb. Med..

[27] Ozioma E.-O.J., Chinwe O.A.N. (2019). Herbal medicines in African traditional medicine. Herb. Med..

[28] Statistics South Africa (2018). Community Survey 2016.

[29] Leedy P.D., Ormrod J.E. (2001). Practical Research: Planning and Research.

[30] Guba E.G., Lincoln Y.S., Denzin N.K. (1994). Handbook of Qualitative Research.

[31] Aworinde O., Olufemi-Aworinde K., Awotunde O., Adeniji A. (2019). Behavior modifying myths practices and effect on health seeking behavior among pregnant Yoruba women, south western Nigeria–a cross-sectional study. Rwanda Med. J..

[32] Zerfu T.A., Umeta M., Baye K. (2016). Dietary habits, food taboos, and perceptions towards weight gain during pregnancy in Arsi, rural central Ethiopia: A qualitative cross-sectional study. J. Health Popul. Nutr..

[33] Bhat R. (2013). Plants of Xhosa people in the Transkei region of Eastern Cape (South Africa) with major pharmacological and therapeutic properties. J. Med. Plants Res..

[34] Makunga N.P., Philander L.E., Smith M. (2008). Current perspectives on an emerging formal natural products sector in South Africa. J. Ethnopharmacol..

[35] Peprah P., Agyemang-Duah W., Arthur-Holmes F., Budu H.I., Abalo E.M., Okwei R., Nyonyo J. (2019). ‘We are nothing without herbs’: A story of herbal remedies use during pregnancy in rural Ghana. BMC Complement. Altern. Med..

[36] Mureyi D.D., Monera T.G., Maponga C.C. (2012). Prevalence and patterns of prenatal use of traditional medicine among women at selected harare clinics: A cross-sectional study. BMC Complement. Altern. Med..

[37] Peltzer K., Phaswana-Mafuya N., Treger L. (2009). Use of traditional and complementary health practices in prenatal, delivery and postnatal care in the context of HIV transmission from mother to child (PMTCT) in the Eastern Cape, South Africa. Afr. J. Tradit. Complement. Altern. Med..

[38] Aziato L., Odai P.N., Omenyo C.N. (2016). Religious beliefs and practices in pregnancy and labour: An inductive qualitative study among post-partum women in Ghana. BMC Pregnancy Childbirth.

[39] M’Soka N.C., Mabuza L.H., Pretorius D. (2015). Cultural and health beliefs of pregnant women in Zambia regarding pregnancy and child birth. Curationis.

[40] Pallivalappila A.R., Stewart D., Shetty A., Pande B., Singh R., McLay J.S. (2014). Complementary and alternative medicine use during early pregnancy. Eur. J. Obs. Gynecol. Reprod. Biol..

[41] Ngoma C.M. (2016). Factors influencing women’s optimum health in Zambia. J. Healthc. Commun..

[42] Beste J., Asanti D., Nsabimana D., Anastos K., Mutimura E., Merkatz I., Sirotin N., Nathan L.M. (2015). Use of Traditional Botanical Medicines During Pregnancy in Rural Rwanda. J. Glob. Health Perspect..

[43] Sychareun V., Somphet V., Chaleunvong K., Hansana V., Phengsavanh A., Xayavong S., Popenoe R. (2016). Perceptions and understandings of pregnancy, antenatal care and postpartum care among rural Lao women and their families. BMC Pregnancy Childbirth.

[44] Yetter G. Roasting and Steaming in Cambodia: Traditional Health Practices for Pregnant Women. https://latitudes.nu/roasting-and-steaming-in-cambodia-traditional-health-practices-for-pregnant-women/.

[45] Begum S., Sebastian A., Kulkarni R., Singh S., Donta B. (2017). Traditional practices during pregnancy and childbirth among tribal women from Maharashtra: A review. Int. J. Commun. Med. Public Health.

